# Super‐high procoagulant activity of gecko thrombin: A gift from sky dragon

**DOI:** 10.1111/cns.14250

**Published:** 2023-05-05

**Authors:** Hao Liang, Xingyuan Zhang, Yuxuan Hou, Kang Zheng, Huifei Hao, Bingqiang He, Hui Li, Chunshuai Sun, Ting Yang, Honghua Song, Rixin Cai, Yingjie Wang, Haiyan Jiang, Lei Qi, Yongjun Wang

**Affiliations:** ^1^ Key Laboratory of Neuroregeneration of Jiangsu and Ministry of Education, Co‐innovation Center of Neuroregeneration Nantong University Nantong PR China; ^2^ Anti‐aging & Regenerative Medicine Research Institution, School of Life Sciences and Medicine Shandong University of Technology Zibo PR China; ^3^ Department of Emergency Medicine Affiliated Hospital of Nantong University Nantong PR China

**Keywords:** nerve, regeneration, reptile, spinal cord, thrombin

## Abstract

**Aims:**

Gecko, the “sky dragon” named by Traditional Chinese Medicine, undergoes rapid coagulation and scarless regeneration following tail amputation in the natural ecology, providing a perfect opportunity to develop the efficient and safe drug for blood clotting. Here, gecko thrombin (gthrombin) was recombinantly prepared and comparatively studied on its procoagulant activity.

**Methods:**

The 3D structure of gthrombin was constructed using the homology modeling method of I‐TASSER. The active gthrombin was prepared by the expression of gecko prethrombin‐2 in 293 T cells, followed by purification with Ni^2+^‐chelating column chromatography prior to activation by snake venom‐derived Ecarin. The enzymatic activities of gthrombin were assayed by hydrolysis of synthetic substrate S‐2238 and the fibrinogen clotting. The vulnerable nerve cells were used to evaluate the toxicity of gthrombin at molecular and cellular levels.

**Results:**

The active recombinant gthrombin showed super‐high catalytic and fibrinogenolytic efficiency than those of human under different temperatures and pH conditions. In addition, gthrombin made nontoxic effects on the central nerve cells including neurons, contrary to those of mammalian counterparts, which contribute to neuronal damage, astrogliosis, and demyelination.

**Conclusions:**

A super‐high activity but safe procoagulant candidate drug was identified from reptiles, which provided a promising perspective for clinical application in rapid blood clotting.

## Graphical abstract

Gecko, also named as sky dragon by Traditional Chinese Medicine, has evolved a rapid coagulation mechanism in response to frequent loss of tail in the natural ecology. Gecko thrombin exhibits super‐high procoagulant activity under various temperature and pH conditions. Meanwhile, this serine protease confers undetectable neurotoxicity, providing a promising perspective in the development of efficient and safe procoagulant drug.

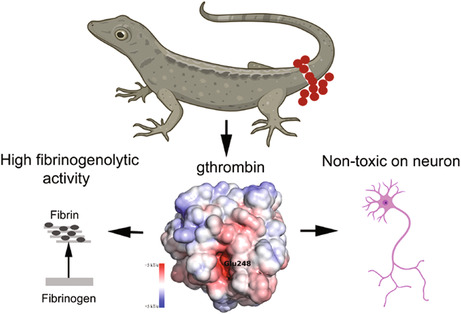

## 1 INTRODUCTION

High efficient procoagulant drug is urgent for immediate blood clotting, as excessive hemorrhage caused by natural disaster, war, or several diseases often results in the death of the subjects. Several species of reptiles, if not all, have evolved a rapid coagulation mechanism that prevents them from excessive bleeding and infection, providing an alternative opportunity for the development of the efficient procoagulant drug. As lower amniotes, reptiles occupy an important evolutionary position in the phylogeny. It is estimated that more than 10,000 reptile species live in the world, being classified into four orders, i.e., *Crocodilia*, *Testudines*, *Squamata*, and *Sphenodontia*.^1^ Some of them (*Crocodilia*) are in huge size that often fights for territory, preys, or even mating rights, while a part of them (*Squamata*) in tiny size becomes preys of other predators. The rapid blood clotting following injury makes them escape from the frequent danger of death. The blood clotting in vertebrates is carried out by cells (thrombocytes or platelets) and a series of thrombin‐mediated protease reactions. Thrombin, a multifunctional serine protease, plays key roles in procoagulant pathways of hemostasis.^2^ It cleaves fibrinogen into fibrin, which is stabilized by factor XIIIa‐catalyzed cross‐links.^3^ Also, thrombin can accelerate coagulation by activation of factor V and factor VIII.^4^, ^5^ Such procoagulant mechanism of thrombin has been shown to be phylogenetically conserved across the vertebrates, though several factors such as FXI and/or FXII are absent from fish and chicken.^6^, ^7^ Conversely, thrombin is able to activate an anticoagulant pathway by binding to thrombomodulin on vascular endothelial cells to promote the activation of protein C.^2^ The relevant enzymatic properties and physiological function of thrombin have been extensively documented in several vertebrates including fish, amphibian, birds, and mammals.^6^, ^8^ However, relevant evidences from reptiles are lacking.

Thrombin is proteolytically generated from its zymogen, prothrombin, by cleavage of factor Xa in the presence of factor Va, calcium ions, and phospholipid.^9^ Prothrombin is biosynthesized by hepatocytes and circulates in the blood, containing a Gla domain, two kringle domains, and a protease domain.^10^ Removal of the Gla domain and two kringle domains from prothrombin by factor Xa will result in the smallest single‐chain precursor to α‐thrombin, the prethrombin‐2. This intermediate product can be prokaryotic and eukaryotic expressed in high yield for preparing active thrombin. However, the catalytic efficacy of prothrombinase for prethrombin‐2 is much lower than for prothrombin.^9^ Ecarin, a snake venom‐derived protease isolated from *Echis carinatus*, has been found to have a specific and high activity in cleavage of prethrombin‐2 to form thrombin.^9^, ^11^

In addition to facilitating hemostasis, thrombin is involved in the regulation of multiple physiological and pathological processes, such as embryonic development, wound healing, inflammation, atherosclerosis, sepsis, and cancer.^12^ However, the protease is currently highlighted for its roles in the central nervous system (CNS), in which it mediates distinct neuronal and glial responses (cytoprotective or cytotoxic) through activation of G‐protein coupled protease‐activated receptors (PARs).^13^, ^14^ High concentration of thrombin is proved to be neurotoxic that can cause vascular disruption,^15^ microglial activation,^16^ synaptic dysfunction,^17^ neuronal damage,^18^ astrogliosis, and demyelination of CNS in pathology.^19^ Many neurological diseases, such as acute ischemic stroke,^20^ intracerebral hemorrhage,^21^ Alzheimer's disease,^22^ Parkinson's disease,^23^ and multiple sclerosis, are linked with aberrant activation of thrombin. Elevated expression of thrombin has been observed in the traumatic spinal cord of mice and contributes to functional decline.^19^ While knockout of PAR‐1 receptor in mice displays improved locomotor recovery and reduces signatures of inflammation and astrogliosis, suggesting the detrimental action of the serine protease in the damaged spinal cord.^19^ However, the pathophysiological functions of reptilian thrombin following CNS insults remain elucidated.

The amniotic gecko, also named as sky dragon in Traditional Chinese Medicine, is able to autotomize its tail once being captured by the predators. The biopsy site rapidly develops a clot of blood for minimal bleeding, which will be lost in 8–14 days following wounding. The animal proceeds to regenerate the lost part of the tail by regrowth of new dermis, skeletal muscle, cartilage, and nerves.^24^, ^25^, ^26^ Notably, wounding‐induced activation of thrombin has no deleterious effects on the regenerating nerve tissues, contrary to those observed in mammals. Such distinct physiological property of gecko thrombin (gthrombin) has provided a promising perspective for developing a safe and efficient procoagulant drug in control of multiple types of acute and chronic hemorrhage, especially for the incidence of CNS bleeding. To quantify the enzymatic activity and assess the neurotoxic effects of gthrombin, we analyzed the characteristics of gecko prothrombin and then prepared the prethrombin‐2 recombinant protein. Subsequently, we acquired the active gthrombin by Ecarin cleavage of prethrombin‐2, followed by measurement of the enzymatic activity, as well as evaluation of its toxic effects on the vulnerable central nerve cells. Our study has revealed a super‐high procoagulant candidate drug with nontoxicity, which has never been characterized before as far as we know.

## 2 METHODS

### 2.1 Gecko model

Adult *Gekko japonicus* was obtained from the Experimental Animal Center of Nantong University. They were fed mealworms ad libitum and were housed in an air‐conditioned room with a controlled temperature (25–28°C) and saturated humidity. Anesthesia was induced by cooling the animals on ice prior to tail amputation. Amputation was performed at the sixth caudal vertebra, based on the special tissue structure present at that position,^25^ by placing a slipknot of nylon thread and pulling gently until the tail was detached, thus mimicking the process of natural defense. All experiments were conducted in accordance with the guidelines of the NIH (Guide for the Care and Use of Laboratory Animals: 1985) and the *Guidelines for the Use of Animals in Neuroscience Research by the Society for Neuroscience*. The experiments were approved according to the *Animal Care and Use Committee of Nantong University and the Jiangsu Province Animal Care Ethics Committee*. All geckos were anesthetized on ice prior to sacrifice.

### 2.2 Cell culture and treatment

PC12 cells were grown in RPMI 1640 media (Invitrogen, Shanghai, China) supplemented with 5% horse serum, 10% (v/v) fetal bovine serum, 50 units/mL penicillin, and 50 μg/mL streptomycin at 37°C in a humidified incubator with 5% CO_2_. PC12 cells were switched to differentiating media (DM; RPMI 1640 with L‐glutamine, 0.2% horse serum, and 100 unit penicillin/100 mg streptomycin) after 24 h. After serum starvation (in DM) for 18 h, cells were exposed to 10 μg/mL thrombin, and alternatively followed by treatment with 50 ng/mL NGF to induce differentiation. The culture of gecko oligodendrocyte cell line Gsn3, and astrocyte cell line Gsn1, was referred to the methods by Wang et al.^27^ The cells were grown in DMEM supplemented with 10% fetal bovine serum at 30°C supplied with 5% CO_2_. The cells with 95% confluency were changed to serum‐free DMEM and stimulated with 0, 5, and 10 μg/mL gthrombin for 24 h, respectively.

### 2.3 Antibodies and reagents

Antibodies against the GFAP (80788S, 1:5000), p‐ERK1/2 (9102S, 1:1000), ERK1/2 (4370S, 1:1000), p‐P38 (4511S, 1:1000), P38 (8690S, 1:1000), p‐JNK (9251S, 1:1000), JNK (9252S, 1:1000) and GAPDH (97166S, 1:1000) were obtained from Cell Signaling Technology. Galactocerebroside (SAB1402780, 1:200) and β‐tubulin (C4585, 1:1000) antibodies were obtained from Sigma. While the secondary antibodies coupled to rabbit HRP (15015, 1:5000) or mouse HRP (15014, 1:5000) were obtained from Proteintech.

Primers for obtaining the full length of gecko prothrombin (gPTM) were: anti‐sense primer 5′‐CCA GCC GGT CAC TCT GCC CTT GTA G‐3′ and sense primer 5′‐GGC AGG TTA TGC TGT TTA GAA AGT‐3′; for cloning gecko prethrombin‐2 (gPre2) into pCDNA3.1(−) vector (Novagen): forward primer 5′‐GAA TTC ACA GCT GCC CAA CAG CGC GAA CTC TTC‐3′ and reverse primer 5′‐AAG CTT TTA ATT TCC ATG CTT CTC TAC AGT C‐3′; for RT‐PCR assays: forward primer 5′‐GCA GCA ATG AAT GAT GAG AG‐3′ and reverse primer 5′‐CAC ATA GCC AAC AAC ATA GC‐3′. Primers for gecko myelin basic protein (MBP): forward primer 5′‐TG ATC CAG GGG GAA GCA GAG‐3′ and reverse primer 5′‐TG TGC CAG TGA GTG CTT CAT‐3′; for gecko proteolipid protein (PLP): forward primer 5′‐GC AGC AAT GAA TGA TGA GAG‐3′ and reverse primer 5′‐CA CAT AGC CAA CAA CAT AGC‐3′; for gecko myelin protein P0 (MPZ): forward primer 5’‐CCT TCA AAT ATG CCT GGG T‐3′ and reverse primer 5’‐CAG CAC AGT CAG CTT GAG AG‐3′; for Taqman probe of EF‐1α: 5′‐TTG GAC AAG CTG AAG GCA GAA CGT G‐3′. All the primers were designed and obtained from Invitrogen.

The EdU DNA Cell Proliferation Kit for the determination of cell proliferation was obtained from Ribobio. The Ni^2+^‐chelating column chromatography for the purification of recombinant proteins was obtained from Sangon. The Omniscript Reverse Transcription Kit for PCR assay was obtained from QIAGEN.

### 2.4 Cloning and analysis of gecko prothrombin

Sequence of gPTM was annotated from genome sequence, which was deposited in the GenBank.^28^ To obtain the full length of gPTM, the primers were designed according to the genome sequences.^28^ Both 5′‐RACE and 3′‐RACE were performed using the SMARTer RACE 5′/3’ Kit (Clontech, Mountain View, CA, USA) according to the manufacturer's instructions.

### 2.5 Sequence analysis and homology modeling

Comparison against the protein database was performed using the PSI‐BLAST network server at the National Center for Biotechnology Information. Multiple protein sequences were aligned using the MegAlign program by the CLUSTAL method in the DNASTAR software package. Phylogenetic tree was constructed using the PHYML implementation of Maximum‐Likelihood, with the GTR (CDS sequences) and JTT (protein sequences) substitution model.

The three‐dimensional structure of gthrombin was generated by the I‐TASSER suite based on the amino acid sequence (XP_015262498.1).^29^ A total of five predicted models were produced by the I‐TASSER, and the one with the highest C‐score was chosen as the final model. The electrostatic potential maps of the proteins were computed using the APBS (Version 3.0).^30^ The results were visualized using the PyMOL (The PyMOL Molecular Graphics System, Version 1.8 Schrödinger, LLC) and VMD (Version 1.9.3).^31^

### 2.6 Preparation of gecko prethrombin‐2 recombinant protein

The open reading frame of gPre2 was amplified from cDNA using ECoR I‐ and Hind III‐linked primers, and the anti‐sense primer was designed to omit the stop codon and enable the translation of the C‐terminal His tag on the pCDNA3.1(−) vector. The pCDNA3.1(−) vector (Novagen) and PCR amplicon were digested with ECoR I and Hind III and ligated. The 293 T cell line was transformed with pCDNA3.1(−)‐gPre2 expression vector and cultured for 48 h at 37°C. Recombinant gPre2 expressed by the cells was harvested by centrifugation at 2500 r/min for 5 min at 4°C. The cells were then disrupted in NTA binding buffer (20 mM Tris–HCl, 500 mM NaCl, and 10 mM imidazole, pH 7.9), and incubated for 30 min in an ice bath. Cell suspension was disrupted by sonication, and centrifuged at 12,000 *g* for 10 min at 4°C. The supernatant was loaded onto a Ni^2+^‐chelating column chromatography (Ni‐NTA SefinoseTM Resin, Sangon Biotech, Shanghai) that had been equilibrated with NTA binding buffer. The C‐terminal His‐tagged recombinant fusion protein was eluted with the elution buffer (20 mM Tris–HCl, 500 mM NaCl, and 500 mM imidazole, pH 7.9) following the manufacturer's instructions. The eluted fusion gPre2 was stored in the stock solution (20 mM Tris, 300 mM NaCl, 10% glycerol, pH 7.5) at −20°C before before converted into thrombin.

### 2.7 Ecarin‐cleaved activation of gthrombin

A total of 0.01, 0.05, 0.1, 0.15, or 0.2 U of Ecarin (Sigma, USA) was added to 3 μg of gPre2 recombinant protein solution, and the mixture was incubated at 37°C for 16 h. Sodium dodecyl sulfate–polyacrylamide gel electrophoresis (SDS‐PAGE) was carried out to examine the cleavage efficiency of Ecarin, and 0.05 U of the snake venom was chosen as work concentration for activation of 3 μg gPre2. The reaction mixture was applied to the Ni^2+^‐chelating column for affinity chromatography, and the gthrombin was purified as per above description.

### 2.8 Enzyme kinetics and fibrinogen‐clotting assay of gthrombin and hthrombin

The enzymatic activity of gthrombin or human thrombin (hthrombin, Sigma, USA) was assayed by using synthetic substrate S‐2238 (Aglyco, Beijing, China). The S‐2238 was dissolved in the 0.01 M PBS, and adjusted to 0.2 mM, 0.4 mM, or 0.6 mM at the final reaction concentration. The release of *p*‐nitroaniline resulting from hydrolysis of S‐2238 was determined in absorbance at 405 nm. Measurement was performed in assay buffer containing 0.01 M PBS at 4, 30 and 37°C in pH 7.0, or at 37°C in pH 6.0, 7.0, and 8.0, respectively, and the reaction was monitored for 2–20 min. Values and standard deviations for Km and *k*cat were calculated from triplicate assays by least‐squares fit to a straight line of a plot of the inverse of the rate of *p*‐nitroaniline release against the inverse of the concentration of S‐2238, using the program LINFIT.

Before clotting, 20 μL (1 μg) of purified gthrombin or hthrombin was added into 150 μL of 0.15 M NaCl in 96‐well plates. Clotting was initiated by mixing 50 μL fibrinogen (final concentration 10 mg/mL) in the well. Clot turbidity was monitored by the spectrometer with absorbance at 550 nm in every 2 min following mixture, and the clotting time was determined from the end of the clot lag period to 90% maximum turbidity.

### 2.9 Quantitative real‐time polymerase chain reaction

Total RNA was prepared with Trizol (Gibco, USA) from gecko blood or Gsn3 cell line. The first‐strand cDNA was synthesized using an Omniscript Reverse Transcription Kit (QIAGEN) in a 20 μL reaction system that contained 2 μg total RNA, 0.2 U/μl M‐MLV reverse transcriptase, 0.5 mM dNTP mix, and 1 μM Oligo‐dT primer. The cDNA was diluted 1:5 before use in the quantitative real‐time polymerase chain reaction (Q‐PCR) assays. Q‐PCR reactions were performed in a final volume of 20 μL (1 μL cDNA template and 19 μL Q‐PCR reaction buffer containing 2.5 mmol/L MgCl_2_, 0.2 mmol/L dNTPs, 0.5 μmol/L anti‐sense and sense primers, 0.4 μmol/L Taqman probe, 0.2 μL DNA polymerase, and 1 × DNA polymerase buffer). The Rotor‐Gene 5 software (Corbett Research, Rotor‐Gene, Australia) was used for real‐time PCR analysis. The reactions were processed using one initial denaturation cycle at 94°C for 5 min, followed by 40 cycles of 94°C for 30 s, 60°C for 30 s, and 72°C for 30 s. Fluorescence was recorded during each annealing step. At the end of each PCR run, the data were automatically analyzed by the system. The full‐length plasmid of gPre2 was used to prepare standard curves. The expression levels of the gecko MBP cDNA were normalized to the endogenous EF‐1α. In addition, a negative control without the first‐strand cDNA was simultaneously carried out.

### 2.10 Western blot analysis

Protein was extracted from cells with a buffer containing 1% SDS, 100 mM Tris–HCl, 1 mM PMSF, and 0.1 mM β‐mercaptoethanol. After centrifugation at 13,000 r/min for 30 min at 4°C, 20 μg of total protein of each sample was loaded into a 10% SDS‐PAGE gel and transferred to PVDF membranes (Millipore Sigma, USA). The membrane was then blocked with 5% nonfat dry milk in TBS containing 0.05% Tween‐20 (TBS‐T) for 1 h, followed by incubation with primary antibodies at 4°C overnight. A further reaction with the second antibody was performed at room temperature for 2 h, and the HRP activity was detected using enhanced chemiluminescence. The membrane was scanned with a ChemiDOC XRS+ Imager (Bio‐Rad, Hercules, CA, USA). The data were analyzed using PDQuest 7.2.0 software (Bio‐Rad). GAPDH was used as an internal control.

### 2.11 Immunostaining of the cells

The permeabilized cells were incubated with monoclonal anti‐β‐tubulin‐Cy3 antibody or polyclonal rabbit anti‐bovine galactocerebroside antibody for 36 h at 4°C. After washing, the cells were stained with the TRITC‐labeled goat anti‐rabbit IgG antibody, Cy3 conjugate (1:400 dilution, Proteintech, USA) overnight at 4°C, followed by a counterstaining with the Hoechst 33342 (1 mg/mL) for 10 min at 37°C. The cells were further mounted on slide glasses with mounting medium and were photographed by a Nikon Diaphot microscope. For quantification of neurite length and Gsn3 process, the neurite or process in each neuron or oligodendrocyte was traced manually, and measured with Photoshop and NIH Image software. A number of over 100 neurons or oligodendrocytes were included in the statistical analysis.

### 2.12 Wound healing assay

Gsn1 cells were seeded into each well of a 12‐well plate and grown to confluent monolayers. The cells were then starved in DMEM supplemented with 0.15 mg/mL of mitomycin C (Sigma, USA) for 12 h, followed by scratching to generate a standardized 500‐mm wound. The cells were incubated at 0–10 μg/mL gthrombin and allowed for further culture at 24 h. Closure of the wound was monitored and photographed at multiple sites. Representative images were captured and analyzed with Wimscratch Quantitative Wound Healing Image Analysis (Wimasis GmbH, Munich, Germany).

### 2.13 Cell proliferation assay

Gsn1 or Gsn3 cells were suspended in the fresh prewarmed cell culture medium and plated at a density of 1 × 10^5^ cells/ml in a 96‐well plate precoated with 0.01% poly‐L‐lysine. After thrombin addition, 50 mM of EdU was added and incubated for an additional 2 h. Finally, the cells were fixed with 4% formaldehyde in PBS for 30 min. The cells were assayed using Cell‐Light EdU DNA Cell Proliferation Kit according to the manufacturer's protocol. Cell proliferation (ratio of EdU^+^ to all cells) was analyzed in randomly selected fields under a DMR fluorescence microscope (Leica Microsystems, Bensheim, Germany). The assays were repeated three times in triplicate.

### 2.14 Measurements of intracellular calcium concentration [Ca^2+^]_i_

Calcium imaging was prepared as described previously.^20^ Cells were loaded with 2 μM Fluo4 AM in DMEM for 30 min at 37°C. The cells were then washed for 20 min in extracellular solution (ECS) before transfer to the chamber for imaging under a microscope (Olympus, BX51) with a 40 × water‐immersion objective. The time course of the changes in fluorescence of Fluo4 AM was obtained at an image of the interval of 5 s with λ emission = 505–525 nm and λ excitation = 488 nm. A low laser power was used to avoid possible fluorescence bleaching. Relative changes in fluorescence intensity were calculated and normalized against the baseline by ∆F/F, where ∆F is the change in fluorescence intensity during stimulation and F is the average fluorescence intensity before stimulation.

### 2.15 Statistical analysis

The statistical significance of the differences between groups was analyzed by one‐way analysis of variance (ANOVA) followed by Bonferroni's post hoc comparison test with SPSS 15.0 (SPSS, Chicago, IL, USA). Prior to statistical analyses, the data sets for each group were tested for normality of distribution using the Kolmogorov–Smirnov test. Statistical significance was set at *p* < 0.05.

## 3 RESULTS

### 3.1 Characterization of gecko prothrombin (gPTH) and construction of three‐dimensional modeling of gthrombin

**FIGURE 1:**
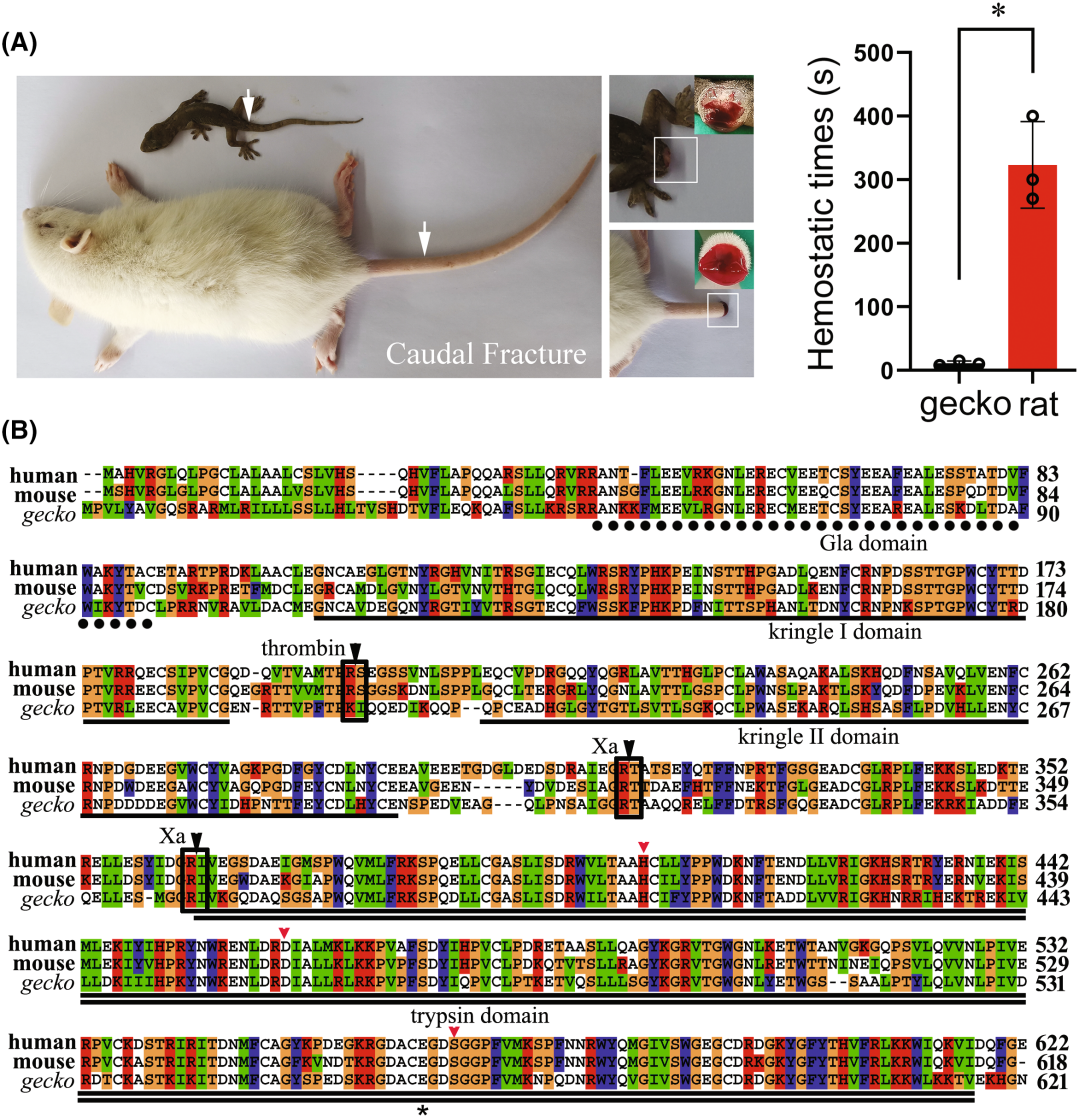
Gross observation of gecko wounding hemostasis and analysis of amino acid sequence of gecko prothrombin. (A) Comparison of gecko and rat natural hemostatic time following tail amputation. The tail of gecko was amputated above 6th, while the rat at 9‐11th caudal vertebra. (B) Multiple alignment of amino acid sequences of gecko prothrombin with those of human and mouse. Each residue in the alignment is assigned a color if the amino acid profile of the alignment at that position meets some minimum criteria specific for the residue type. Gaps introduced into sequences to optimize alignment are represented by dashes. The Gla domain, kringle I domain, kringle II domain, and trypsin domain are indicated by dot line, line, or double line, respectively. The potential cleavage sites by coagulation factor Xa and thrombin are boxed and indicated by the black arrowhead. The conserved catalytic residues, His91, Asp147, and Ser251 (numbering from the amino terminus of the light chain), are indicated by the red arrowhead. The Glu248 for gecko and Glu251 for human, around which the negative electrostatic potentials are analyzed, are indicated by the asterisks. Prothrombin sequences of gecko (XP_015262498), human (AAC63054), and mouse (NP_034298) are obtained from GenBank.

To understand the procoagulant role of gthrombin, a gross observation was made to compare the natural hemostatic time between gecko and rat following their tail amputation. The preliminary timing of stop bleeding at wounding site demonstrated that gecko cost less time for hemostasis than the rat did (Figure 1A). Therefore, the characteristics of gPTH sequences were analyzed by bioinformatics. The gPTH was annotated from genome sequence and deposited in the GenBank under accession number XP_015262498. Multiple alignments indicated that gPTH shared 60.9% and 61.0% sequence identity with the homologs from human and mouse, respectively, with a conserved gamma‐carboxy glutamic acid (Gla) domain, kringle I domain, kringle II domain, and catalytic domain (trypsin domain) (Figure 1B). Three cleavage sites in the mammalian prothrombin, one by thrombin and two by factor Xa (FXa), contained only two FXa cleavage sites of them in the gPTH, but this may not influence the processing of the active thrombin.^3^ Further analysis of representative reptile prothrombin sequences showed that gPTH, together with that of snake, evolved a distinct characteristic by lack of thrombin cleavage site (Figure S1).

**FIGURE 2:**
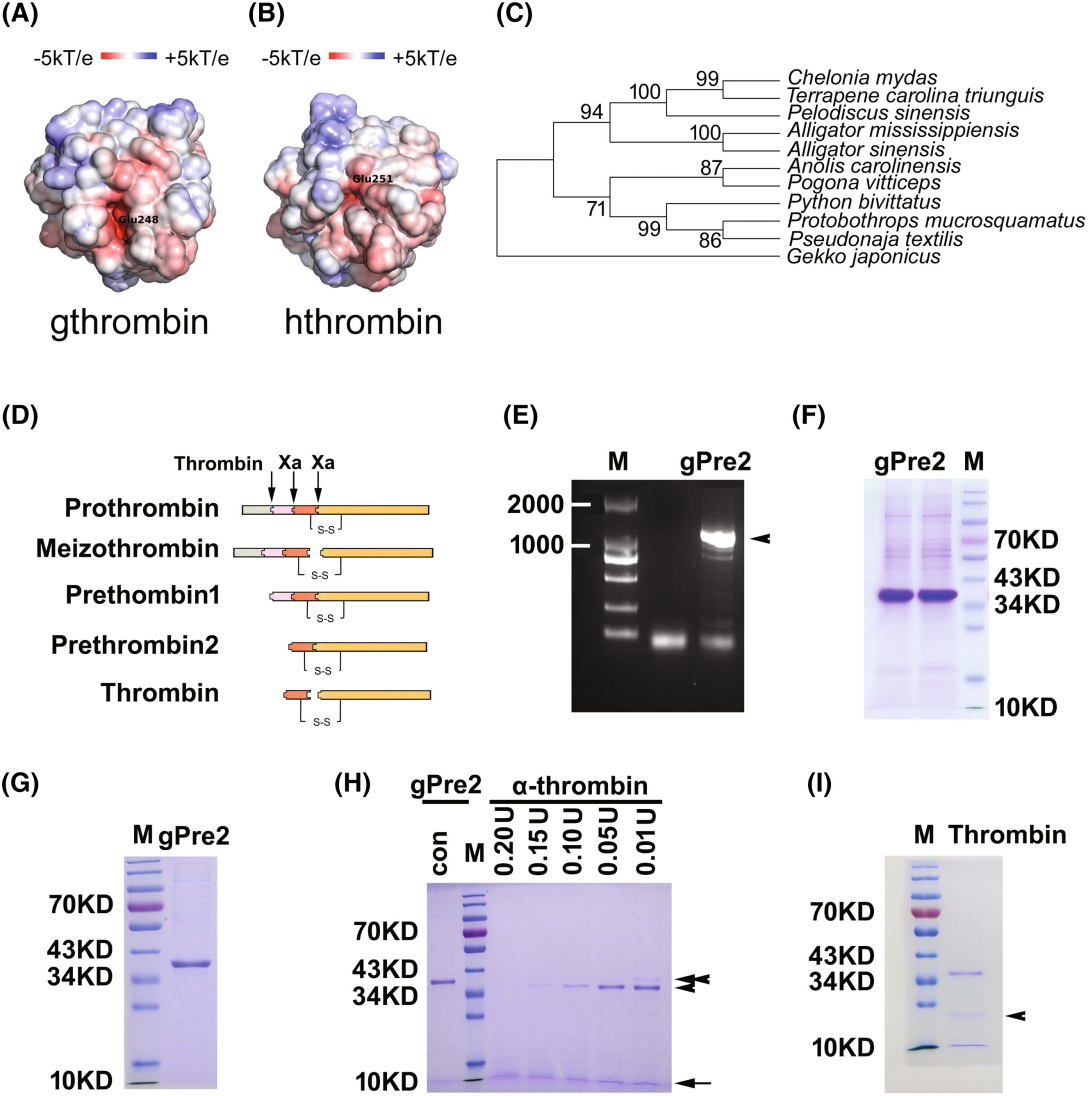
Electrostatic potential and phylogenic analysis of gthrombin, and preparation of the recombinant protein. (A and B) Electrostatic potentials of gecko and human thrombin mapped on the solvent‐accessible surfaces. The red and blue regions represent negative and positive electrostatic potentials, respectively. (C) Phylogenetic tree of prethrombin‐2 sequences annotated from prothrombin of gecko and other representative reptiles constructed by the neighbor‐joining method within the package PHYLIP 3.5c. Bootstrap majority consensus values on 1000 replicates are indicated at each branch point in percent. Prothrombin sequences obtained from GenBank are *Gekko japonicus* (XP_015262498), *Alligator mississippiensis* (XP_006261451), *Alligator sinensis* (XP_006026972), *Anolis carolinensis* (XP_003214643), *Chelonia mydas* (XP_007069796), *Pelodiscus sinensis* (XP_006117365), *Pogona vitticeps* (XP_020654675), *Protobothrops mucrosquamatus* (XP_015679609), *Pseudonaja textilis* (XP_026563591), *Python bivittatus* (XP_007421832), and *Terrapene carolina triunguis* (XP_024070737). (D) Illustration of prothrombin activation. (E) Agarose gel electrophoresis of gecko prethrombin‐2 (gPre2). Arrowhead indicates the according bands of PCR products. (F) The recombinant gPre2 expressed by 293 T cells. (G) The purified recombinant gPre2. (H) 3 μg of gPre2 was subjected to activation by 0–0.20 U of Ecarin in the reaction buffer. Arrowhead indicates the size of B chain of gthrombin, while arrow indicates A chain. Tandem arrowhead indicates the size of gPre2. (I) The purified gthrombin. Arrowhead indicates an unidentified band, which was further analyzed by mass spectrometry to be a degradation product of gthrombin. M, Marker.

As was previously reported, the absolute conservation of the residues that constitute the catalytic apparatus, His91, Asp147, and Ser251 (numbering from the amino terminus of the light chain), was seen in this alignment.^32^ Structurally, these functional catalytic residues are located in an equatorial cleft, which is involved in the specificity of the binding substrates.^33^ Several lines of evidence illustrate that thrombin is allosterically regulated by Na^+^, and the binding of Na^+^ exhibits increased catalytic properties relative to the Na^+^‐free form due to subtle structural changes.^34^ To shed light on the flexibility of gthrombin in the attraction of Na^+^ that is enzymatic activity‐relevant, we compared three‐dimensional structure (3D) and electrostatic potentials of gecko and human thrombin (hthrombin). The 3D modeling of gthrombin was constructed using the homology modeling method according to the procedure of I‐TASSER. The hthrombin structures, PDB codes 4HZH,^35^ 1JWT,^36^ and 1MKW,^37^ were chosen as templates from the PDB library based on the structural similarity. Among the five predicted models, the one with a C‐score value of 0.24 was selected as the final model, where the C‐score was a confidence score for estimating the quality of predicted models by I‐TASSER. The C‐score was in a range between −5 and 2, and generally, a higher C‐score value signifies a model with higher confidence. The predicted gthrombin structure was thus in good quality for further analysis. To address the electrostatic potentials of gthrombin and hthrombin, they were further computed and mapped on the solvent‐accessible surfaces. By using the same range, the electrostatic potential distributions of the two proteins were investigated. As shown in Figure 2A,B, the red regions in the two proteins, corresponding to the negative electrostatic potentials, were around the Glu residues (Glu248 and Glu251 for gecko and human, respectively). Intriguingly, gthrombin showed a lower electrostatic potential than that of the hthrombin, which may lead to more potent activity.

To gain an insight into the evolutionary relationships of reptile thrombin, we analyzed sequences of prethrombin‐2 from representative reptiles including turtles, crocodiles, snakes, and lizards. The phylogenetic tree demonstrates that gthrombin is evolutionarily primitive comparing with those of other reptiles (Figure 2C), indicating an ancient physiological role of gthrombin during the evolution of reptiles.

### 3.2 Expression and purification of gecko prethrombin‐2

To define the physiological roles of gthrombin, the gecko prethrombin‐2 (gPre2) was cloned and ligated into pCDNA3.1(−) plasmid following digestion with ECoR I and Hind III (Figure 2D,E). The recombinant plasmid pCDNA3.1(−)‐gPre2 was expressed in 293 T cells. Following cell culture for 48 h at 37°C, the recombinant gPre2 protein in the cells was purified by Ni^2+^‐chelating column for affinity chromatography (Figure 2F,G). The C‐terminal His‐tagged recombinant fusion protein was verified by SDS‐PAGE, and a distinct band with the approximate molecular weight of 39.0 kDa was detected (Figure 2F,G).

### 3.3 Preparation of recombinant gthrombin

The purified recombinant gPre2 was subsequently subjected to activation by the addition of different concentrations of snake venom‐derived Ecarin. At the ratio of 0.05 U of Ecarin to 3 μg of gPre2, the gPre2 was completely cleaved (Figure 2H). The gthrombin produced in an enlarged scale was highly purified by Ni^2+^‐chelating affinity chromatography, and was preserved in the 0.01 M PBS at a final concentration of 0.284 mg/mL (Figure 2I). An unknown product was simultaneously detected in the samples, which was electrophoretically collected for mass spectrometry analysis (Figure 2I). Results demonstrated that the unidentified band was derived from degradation of gthrombin (Figure S2).

### 3.4 The gthrombin exhibits higher enzymatic activities than those of hthrombin

**FIGURE 3:**
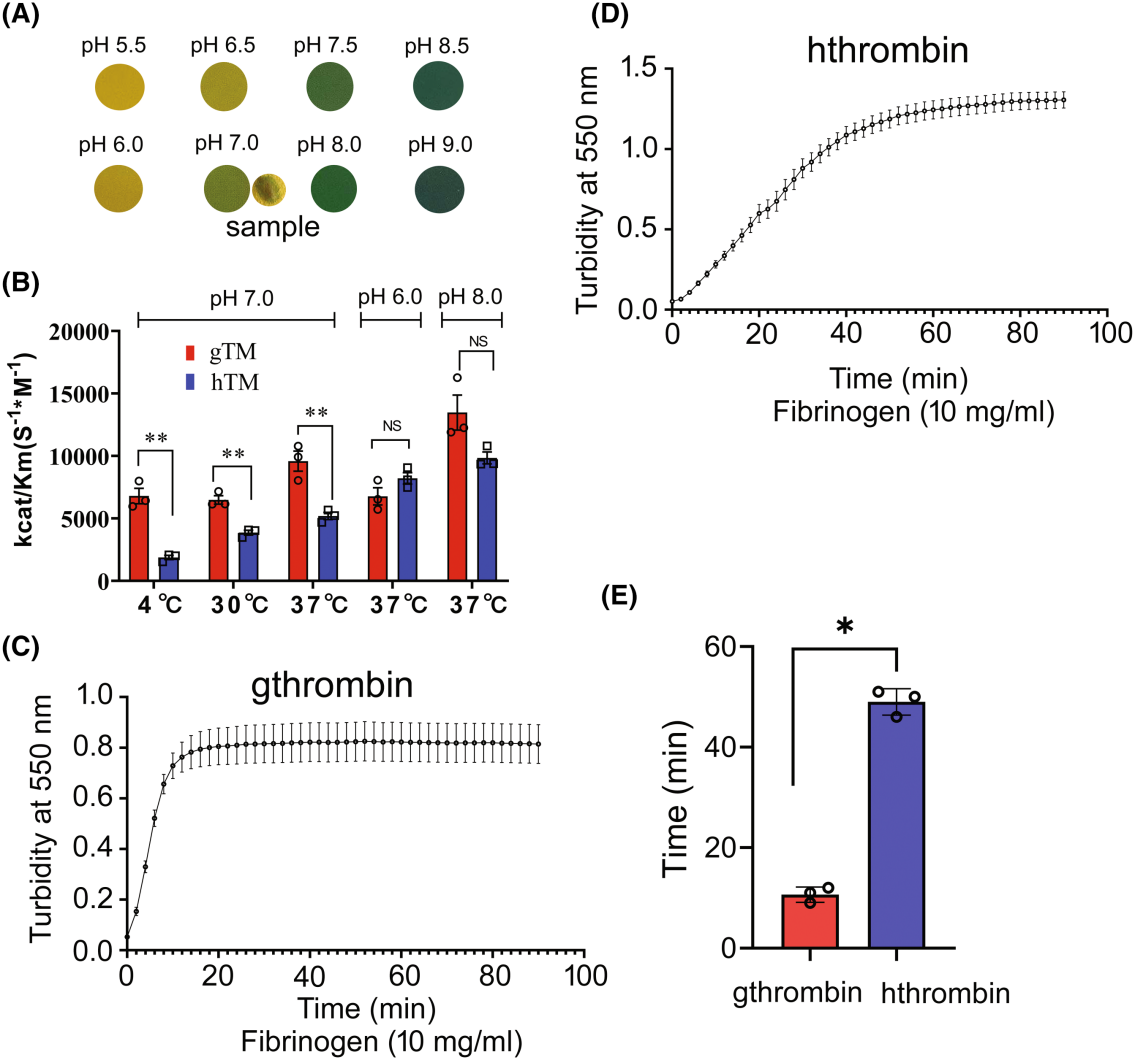
Measurement of enzymatic activity of gthrombin and hthrombin. (A) Examination of tissue fluid pH value following gecko tail amputation. The pH value of the sample was indicated at pH 7.0. (B) The enzymatic activity (*k*cat/Km) of gthrombin and hthrombin was assayed using synthetic substrate S‐2238 at 4, 30, and 37°C at pH 7.0, and pH 6.0, 7.0, and 8.0 at 37°C, respectively. (C and D) Turbidity assays on human fibrin clots at 10 mg/mL fibrinogen with 1 μg gthrombin (C) and hthrombin (D) in every 2 min following initiation. (E) Comparative analysis of clotting time between gthrombin and hthrombin. The clotting time was determined from the end of the clot lag period to 90% maximum turbidity. All assays were carried out in triplicate. Data are represented as mean ± SEM (*p* < 0.01).

To analyze the enzyme kinetics of gthrombin, the enzymatic activities of gthrombin at different pH values or temperatures were assayed by hydrolysis of synthetic substrate S‐2238. As the pH value of tissue fluid following gecko tail amputation is near neutral (Figure 3A), the enzymatic parameters of gthrombin at 4, 30, and 37°C were firstly determined at pH 7.0. As shown in Figure 3B, the maximal enzymatic activity (*k*cat/Km) of gthrombin at pH 7.0 was observed at 37°C, which was significantly higher than that of human. However, the highest catalytic efficiency of gthrombin was detected at alkalescency condition (Figure 3B). The results indicate that the optimal condition for gthrombin action is in weak alkalinity at 37°C.

To examine the fibrinogenolytic action of thrombin, the fibrinogen‐clotting assay was performed by mixing 1 μg gthrombin or hthrombin with 200 μL of 10 mg/mL fibrinogen in 0.15 M NaCl solution. Clot turbidity was monitored in every 2 min, and the clotting time was determined from the end of the clot lag period to 90% maximum turbidity. Results showed that the clotting time of fibrinogen by gthrombin was significantly shorter than that by hthrombin (Figure 3C–E). The data indicate that gthrombin has higher enzymatic activities in clot formation than those of hthrombin.

### 3.5 Nontoxic effects of gthrombin on neuronal differentiation and elongation of neurites

**FIGURE 4:**
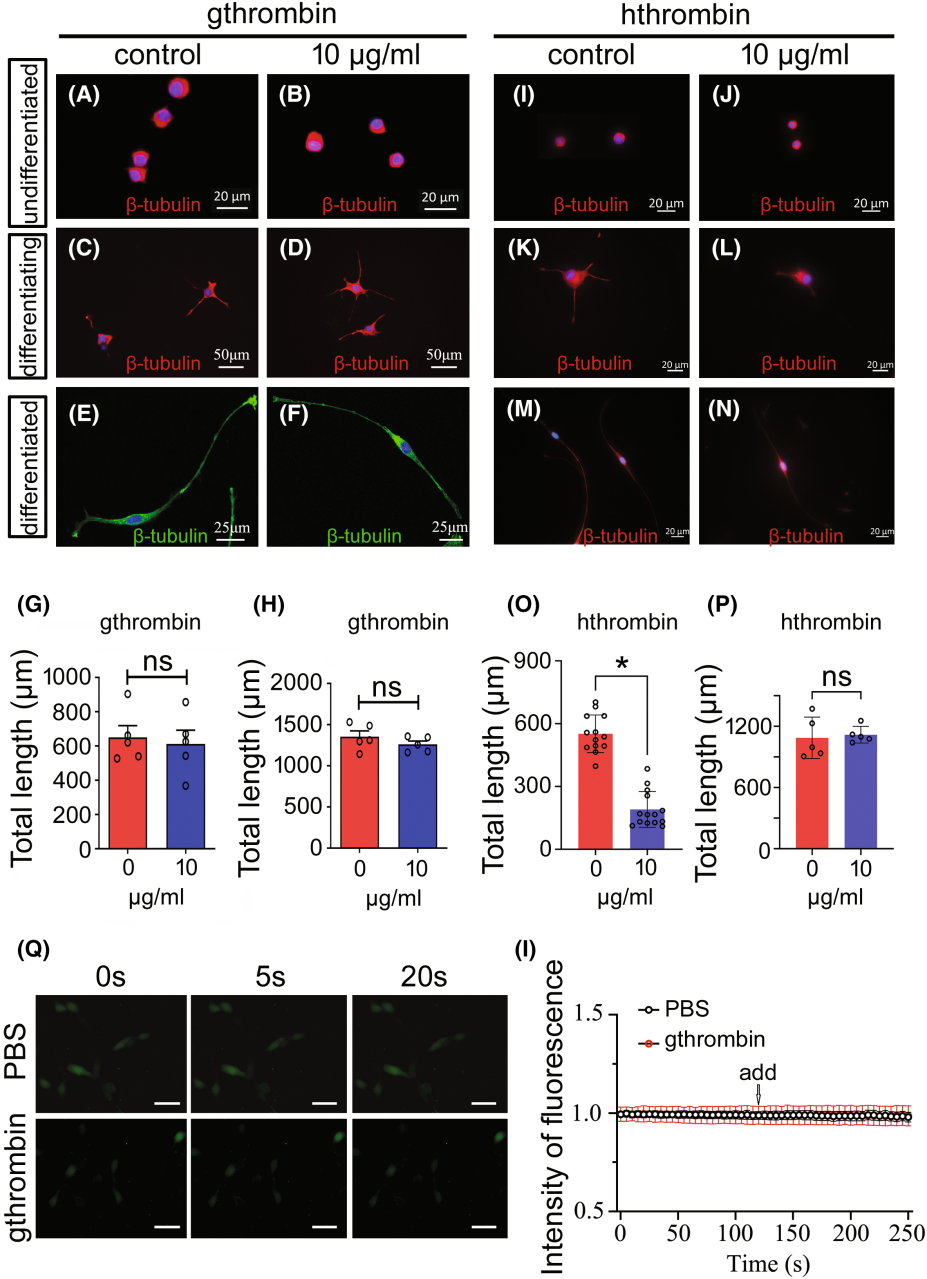
Effects of gthrombin on the differentiation and neurite elongation of PC12 cells. (A and B) Effects of 10 μg/mL gthrombin on the undifferentiated PC12 cells following treatment for 24 h. (C and D) Effects of 10 μg/mL gthrombin on the neurite elongation of differentiating PC12 cells. The cells were stimulated with 10 μg/mL gthrombin for 24 h, followed by incubation at 50 ng/mL NGF for 48 h. (E and F) Effects of 10 μg/mL gthrombin on the neurite elongation of differentiated PC12 cells following treatment for 24 h. (G) and (H) are statistical analyses of (C and D) and (E and F) from five fields each 20 cells. (I–N) The effects of equivalent hthrombin on the undifferentiated (I and J), differentiating (K and L), and differentiated PC12 cells (M and N). (O) and (P) are statistical analyses of (K and L) and (M and N) from 5 to 14 fields each 20 cells. Data are represented as mean ± SEM (*p* < 0.05). Scale bars, 20 μm in (A and B), and (I–N); 50 μm in (C and D); 25 μm in (E and F). (Q) Differentiated PC12 cells were treated with 5 μM Fluo4‐AM for 30 min, followed by the addition of 10 μg/mL of gthrombin. The fluorescence intensity of intracellular free calcium concentration in PC12 cells was recorded every 5 s. (I) The original record of changes in calcium concentration in individual neurons (by ratio of gthrombin/PBS).

Thrombin has been shown to affect neuronal growth and intracellular Ca^2+^ homeostasis.^38^ To address the potential effects of gthrombin on the cellular events of neurons, we cultured PC12 cells, the cell line derived from rat pheochromocytoma with physiological and biochemical functions close to those of neurons, and examined their differentiation and elongation of neurites following cell treatment with gthrombin. Results showed that the addition of 10 μg/mL gthrombin in the serum‐free medium did not significantly induce the differentiation of PC12 cells (Figure 4A,B). When the cells were stimulated with 10 μg/mL gthrombin for 24 h, followed by inducing cell differentiation with 50 ng/mL NGF for 48 h, the total length of neurites showed an undetectable difference comparing with the control, indicating that gthrombin is inefficient in mediating the differentiation of neurons (Figure 4C,D,G). Similarly, treatment of 10 μg/mL gthrombin on differentiated neurons for 24 h did not impact the elongation of neurites (Figure 4E,F,H). However, the parallel experiments on equivalent hthrombin demonstrated that the human‐derived serine protease was able to significantly inhibit the differentiation of PC12 cells induced by NGF, though had undetectable effects on the neurite length of the differentiated neurons (Figure 4I–P). The data indicate nontoxic effects of gthrombin on neurons in different developmental stages.

Thrombin induces a transitory dose‐dependent increase in intracellular free calcium concentration that influences neuronal function.^39^ To understand the effects of gthrombin on the concentration of intracellular Ca^2+^, the differentiated PC12 cells were treated with 5 μM Fluo4‐AM for 30 min, followed by exposure to 10 μg/mL gthrombin. The amplitude of calcium response was measured spectrophotometrically at every 5 s. Results showed that gthrombin failed to change the intracellular Ca^2+^ concentration at all times recorded (Figure 4Q,I).

### 3.6 The gthrombin cannot induce the astroglial reaction of gecko

**FIGURE 5:**
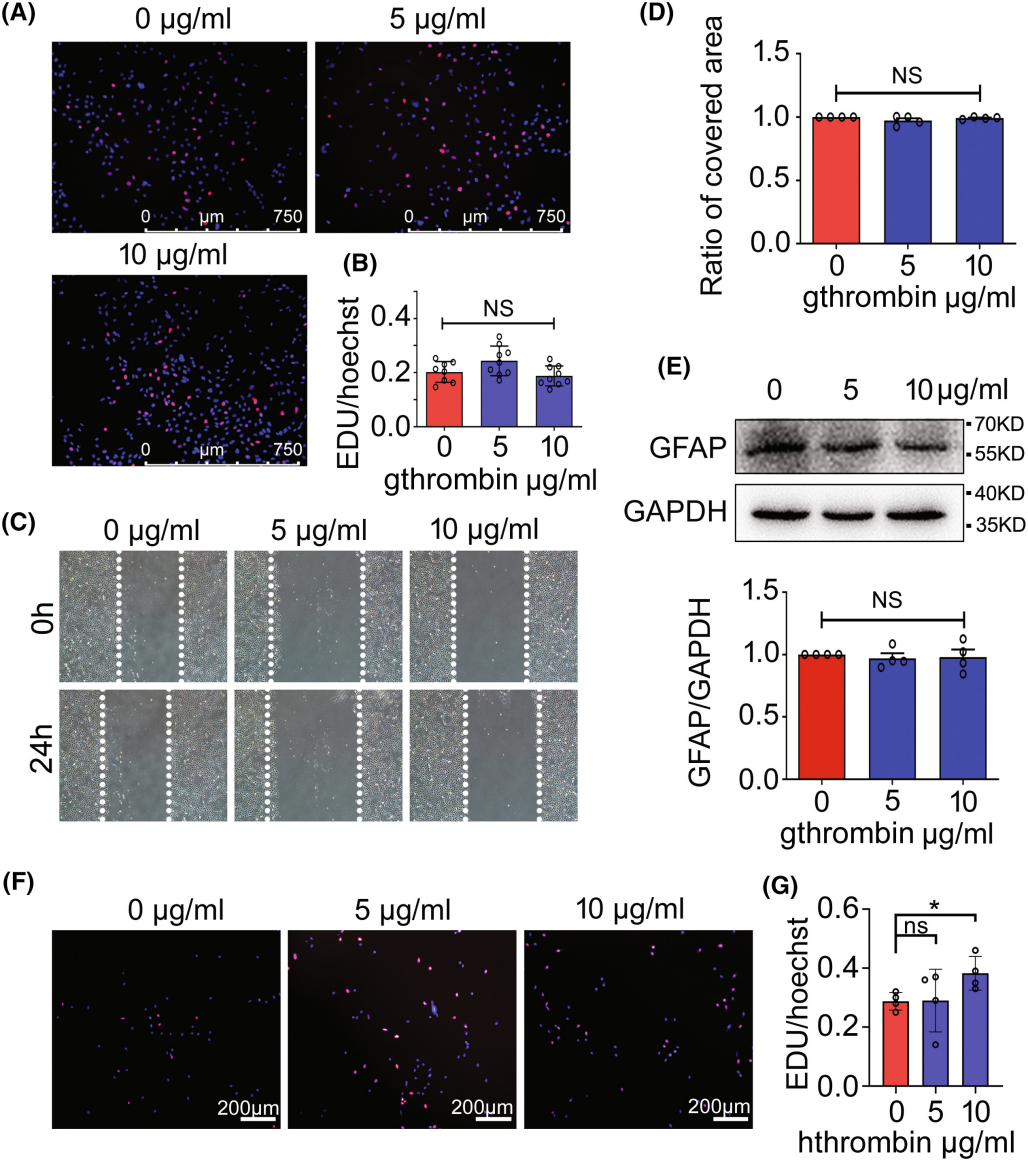
Effects of gthrombin on the cellular events of gecko astrocytes. (A) EdU assay of gecko astrocyte cell line Gsn1 following treatment with 0–10 μg/mL gthrombin for 24 h. (B) Statistical analysis of (A). (C) Wound healing assay of Gsn1 following 0–10 μg/mL of gthrombin treatment for 24 h. The cells were pretreated with 0.15 mg/mL of mitomycin C for 12 h before wound scratching. (D) Statistical analysis of (C). (E) Western blot analysis of GFAP after Gsn1 was treated with 0–10 μg/mL gthrombin for 24 h. (F) EdU assay of Gsn1 following treatment with 0–10 μg/mL hthrombin for 24 h. (G) Statistical analysis of (F). Data are represented as mean ± SEM (*p* < 0.05). Scale bars, 750 μm in (A); 200 μm in (F).

Thrombin has been found to induce astroglial reaction via activation of PARs that are either cytoprotective or cytotoxic.^13^, ^40^ To examine the effects of gthrombin on the cell events of astrocytes from gecko spinal cord, the astrocyte cell line (Gsn1) was treated with 0–10 μg/mL gthrombin for 24 h. The results demonstrated that the gthrombin neither influenced the cell proliferation measured by EdU assay (Figure 5A,B), nor the migration of the astrocytes detected by wound scratching (Figure 5C,D). Western blot analysis revealed that GFAP protein levels in astrocytes were unaffected by the stimulation of gthrombin (Figure 5E). Contrarily, the hthrombin was able to promote the proliferative response of astrocytes (Figure 5F,G), in consistency with those of other mammals.^40^ The data indicate that the gthrombin is not able to induce the astroglial reaction of gecko.

### 3.7 High dose of gthrombin has unfavorable effects on oligodendrocyte function through inhibiting phosphorylation of ERK1/2

**FIGURE 6:**
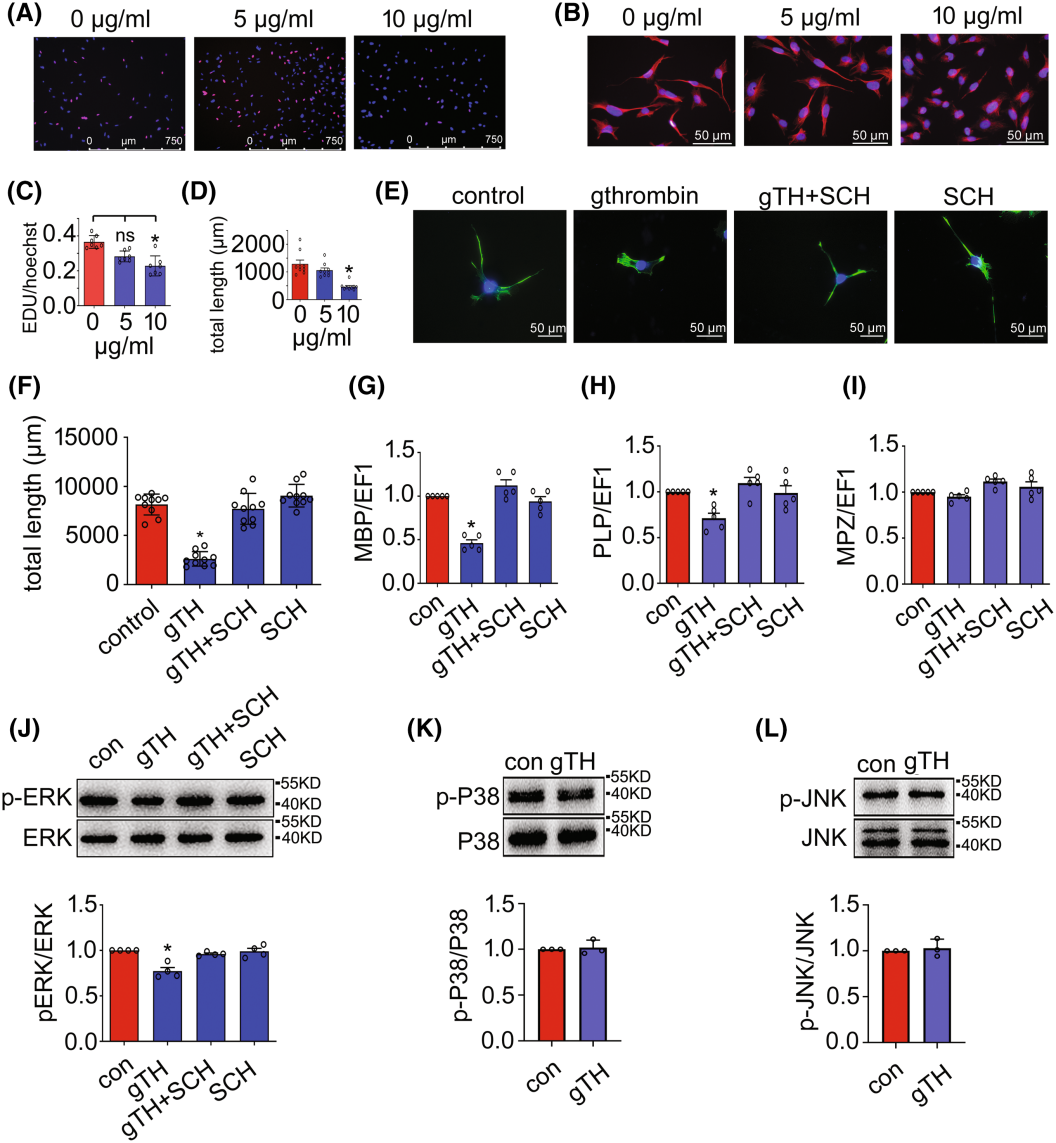
Toxic effects of gthrombin on the gecko oligodendrocytes. (A) EdU assay of gecko oligodendrocyte cell line Gsn3 following treatment with 0–10 μg/mL gthrombin for 24 h. (B) Effects of 0–10 μg/mL gthrombin on the process elongation of Gsn3. (C and D) Statistical analyses of (A) and (B). (E) Exposure of the cells to 500 nM PAR‐1 inhibitor SCH79797 for 24 h attenuated the inhibitory effects of 10 μg/mL gthrombin. (F) Statistical analysis of (C) from 10 fields each 20 cells. Data are represented as mean ± SEM (*p* < 0.05). Scale bars, 750 μm in (A); 50 μm in (B and E). (G–I) RT‐PCR determination of myelination‐related genes including MBP, PLP, and MPZ following oligodendrocyte treatment with 10 μg/mL gthrombin for 24 h. (J–L) Western blot analysis of the phosphorylated ERK, P38, and JNK following Gsn3 treatment with 10 μg/mL gthrombin for 24 h. Experiments were performed in triplicate. Data are represented as mean ± SEM (*p* < 0.05).

Thrombin receptor PAR‐1 has been assumed to be a key suppressor of developmental myelination through regulation of ERK1/2 and AKT signaling.^41^ To test the effects of gthrombin on the oligodendrocytes, gecko oligodendrocyte cell line Gsn3 was incubated in the serum‐free medium containing 0–10 μg/mL of gthrombin for 24 h. An assay of CCK‐8 revealed that gthrombin at a dose of 10 μg/mL significantly decreased the cell viability (Figure S3). The serine protease at a dose of 10 μg/mL also reduced the proliferation of Gsn3, as well as the elongation of processes (Figure 6A–D), which were rescued by the addition of 500 nM of PAR‐1 inhibitor SCH79797 (Figure 6E,F). Such roles of gthrombin on oligodendrocytes were consistent with those of hthrombin, as were examined by the parallel experiments (Figure S4). The transcription of myelin basic protein (MBP) and PLP in Gsn3 was remarkably decreased following cell treatment of 10 μg/mL gthrombin (Figure 6G–I). To elucidate the involved signaling cascade(s) mediated by gthrombin, the activation of ERK1/2 responsible for the regulation of myelin development was then examined. Results showed that gthrombin at a dose of 10 μg/mL decreased the phosphorylation levels of ERK1/2 in Gsn3 (Figure 6J–L). These findings indicate that excessive activation of gthrombin results in unfavorable effects on the oligodendrocyte function of gecko.

## 4 DISCUSSION

Several species of reptiles undergo many times of appendage loss or body injury over their lifetimes and exhibit differential regenerative ability.^42^, ^43^ They have evolved a self‐defense mechanism to protect them from lethal infection by rapid hemostasis and wound healing. A high activity of thrombin is therefore essential for blood coagulation in diverse ecological niches. Many species of lizard including geckos are able to repeatedly regenerate the lost tail in order to adapt their ecological environment.^43^ Unveiling the unique physiological properties of thrombin from these amazing animals will speed up the progress in the development of the procoagulant drugs applicable in chronic hemorrhage. In the present study, we demonstrated that the gthrombin manifested a higher enzymatic activity comparing with that of hthrombin under various conditions, suggesting an adaptive evolution of the reptile in response to the predation pressures. Interestingly, thrombin of the poikilothermal gecko was able to maintain high enzymatic activity at different temperatures, indicating the biological significance of the gthrombin in blood clotting at diverse ecology. Construction of 3D modeling predicted that gthrombin had a lower electrostatic potential around Glu251 than that of the hthrombin, a property favorable for enhanced catalytic activity by binding of Na^+^. However, the conclusion remains further clarified by overall screening those of other reptilian thrombin proteins.

In addition to the central role in hemostasis, thrombin also contributes to the neuropathological progression of CNS, especially in the brain.^44^ The serine protease is produced immediately after hemorrhage or breakdown of blood–brain (spinal cord) barrier. Meanwhile, the parenchymal cells in the CNS are also important producers in response to the injury.^45^, ^46^, ^47^ A low concentration of thrombin (from 50 pM to 100 nM) has been proposed to be neuroprotective by inducing intracellular Ca^2+^ spikes.^45^, ^48^ However, the serine protease will cause neuronal cell death by inducing a sustained Ca^2+^ elevation at the high concentration.^47^, ^49^ In the present study, we determined the viability of PC12 cells incubated at 0–20 μg/mL (equivalent to 584 nM) of gthrombin, which showed nontoxic effects on the cells. The 10 μg/mL of gthrombin also did not impact on the neuronal differentiation, elongation of neurites, and alteration of intracellular Ca^2+^, suggesting a unique physiological property of gthrombin in the balance of hemostasis and regeneration of CNS.

Astrocytes are one of the predominant cell types in the CNS that express PAR‐1, PAR‐3, and PAR‐4 receptors.^50^ Thrombin induces morphological changes and proliferative reaction of astrocytes through proteolytic activation of PAR‐1 signaling.^13^, ^51^, ^52^ These astroglial reactions responding to thrombin stimulation are associated with the formation of glial scar, which will impede functional recovery of the injured CNS in the mammals.^13^ It is generally recognized that the “real” stellate astrocytes are only present in amniotes,^53^ despite several debates.^54^ We have previously demonstrated that gecko can regenerate the spinal cord following injury without evoking astrocytic responses.^55^, ^56^ Here, we also displayed that gthrombin was inefficient in regulating cellular events of astrocytes, recapitulating the in vivo observation in the severed spinal cord of gecko.

Thrombin‐conveyed activation of PAR‐1 generates profound effects on myelination of the CNS. Deletion of PAR‐1 has been found to promote differentiation of oligodendrocyte progenitor cells (OPCs), onset of axon ensheathment, and myelin thickness in adult mouse,^41^, ^57^ indicating that thrombin is a negative regulator of myelination following CNS injury. We demonstrated that 10 μg/mL of gthrombin significantly decreased the proliferation and elongation of oligodendrocyte processes through ERK signaling, suggesting the conserved function of the serine protease in negative regulation of the myelination during the amniotic evolution. It is noteworthy that gecko can spontaneously regenerate the spinal cord composed of ependymal lining and descending axons without associated DRGs following tail amputation.^24^, ^26^, ^58^ Amputation‐induced activation of the gthrombin does not influence the regenerating cord of the gecko. The most possibility attributes to limited bleeding controlled by muscle contracting at a unique fracture plane.^59^ It is estimated that 1 mL of whole blood can only produce about 1–2 nM of thrombin,^48^ which is insufficient in affecting myelination of the oligodendrocytes. There are several concerns regarding the adverse effects of gthrombin on the CNS myelin, which impede its potential application as a procoagulant drug. In the present study, we showed that gthrombin at a dose of 5 μg/mL (146 nM) equivalent to at least 70 mL of whole blood,^48^ made no effects on the cell events of oligodendrocyte, suggesting the less toxicity of the serine protease on the CNS myelin. However, a long‐term in vivo experiment of gthrombin is indispensable for evaluating its exact impact on myelination under physiological and pathological conditions.

In conclusion, the active thrombin has successfully been prepared from gecko, which exhibits super‐high procoagulant activity under various temperature and pH conditions. The gthrombin has evolved to the low neurotoxicity on the vulnerable central nerve cells so as to promote hemostasis with safety.

## AUTHOR CONTRIBUTIONS

Yongjun Wang designed this work. Yongjun Wang wrote the paper. HL and XZ performed the experiments. Yongjun Wang, Hao Liang, Xingyuan Zhang, Yuxuan Hou, Kang Zheng, Huifei Hao, Bingqiang He, Hui Li, Chunshuai Sun, Ting Yang, Honghua Song, Rixin Cai, Yingjie Wang, Haiyan Jiang, and Lei Qi analyzed the data. All authors have approved the present version of the manuscript and have agreed to be accountable for all aspects of the work regarding questions related to the accuracy or integrity of any part of the work.

## CONFLICT OF INTEREST STATEMENT

The authors declare no competing interests.

## Supporting information


Figure S1.

Data S1.

## Data Availability

The data used to support the findings of this study are available from the corresponding author upon request.
