# Supplementary material for: Super‐high procoagulant activity of gecko thrombin: A gift from sky dragon
Source: CNS Neurosci Ther. 2023 May 5;29(10):3081–93. doi: 10.1111/cns.14250 (PMC10493662; doi:10.1111/cns.14250)
Supplement: Supplementary file 1 — Figure S1. [file CNS-29-3081-s001.docx]

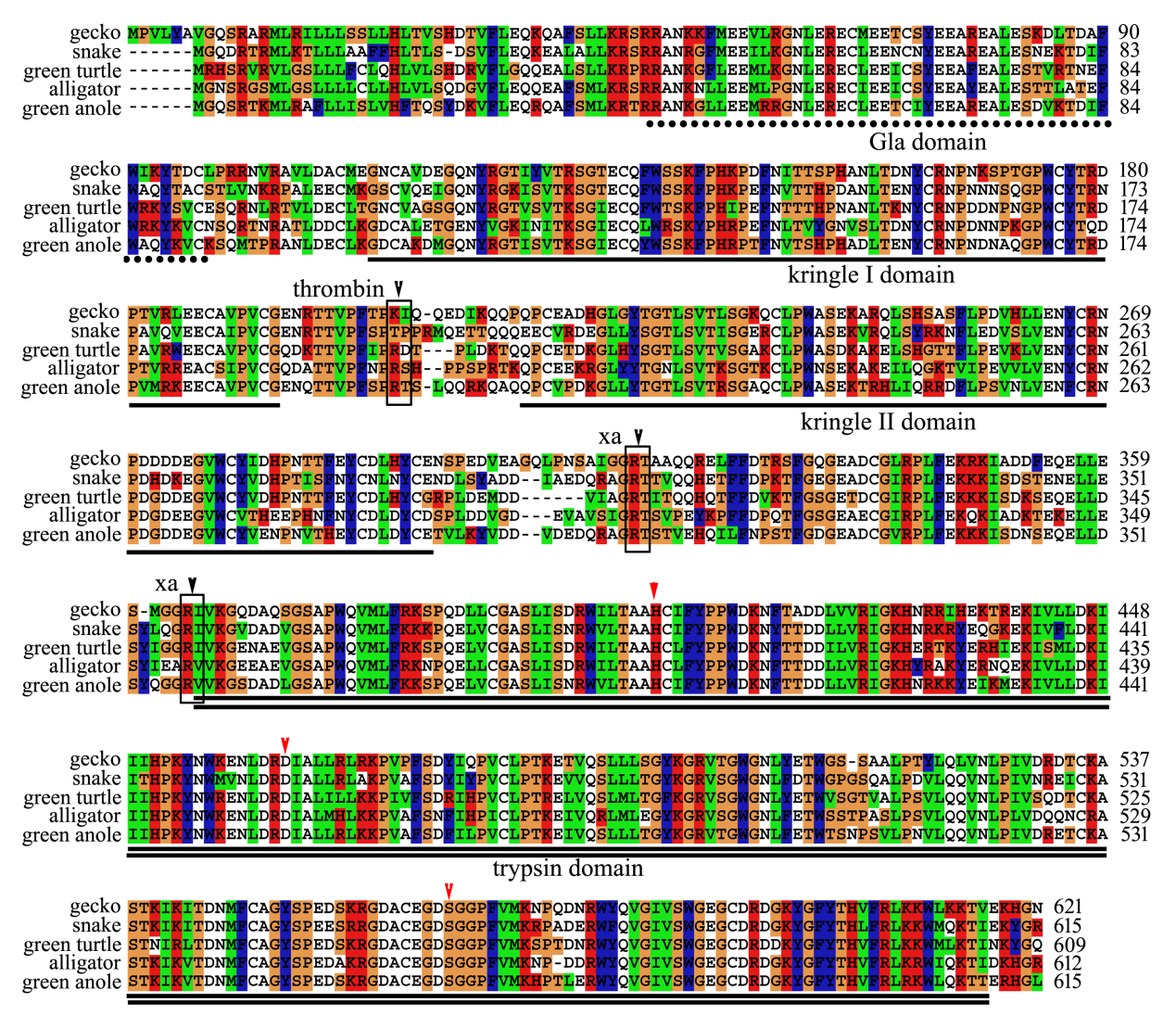


**Fig. S1** Multiple alignment of amino acid sequences of gecko prothrombin with those of representative reptiles. Each residue in the alignment is assigned a color if the amino acid profile of the alignment at that position meets some minimum criteria specific for the residue type. Gaps introduced into sequences to optimize alignment are represented by dashes. The Gla domain, kringle I domain, kringle II domain and trypsin domain are indicated by dot line, line or double line, respectively. The potential cleavage sites by coagulation factor Xa and thrombin are boxed and indicated by the black arrowhead. The conserved catalytic residues, His91, Asp147 and Ser251 (numbering from the amino terminus of the light chain), are indicated by the red arrowhead. Prothrombin sequences referred to figure2.


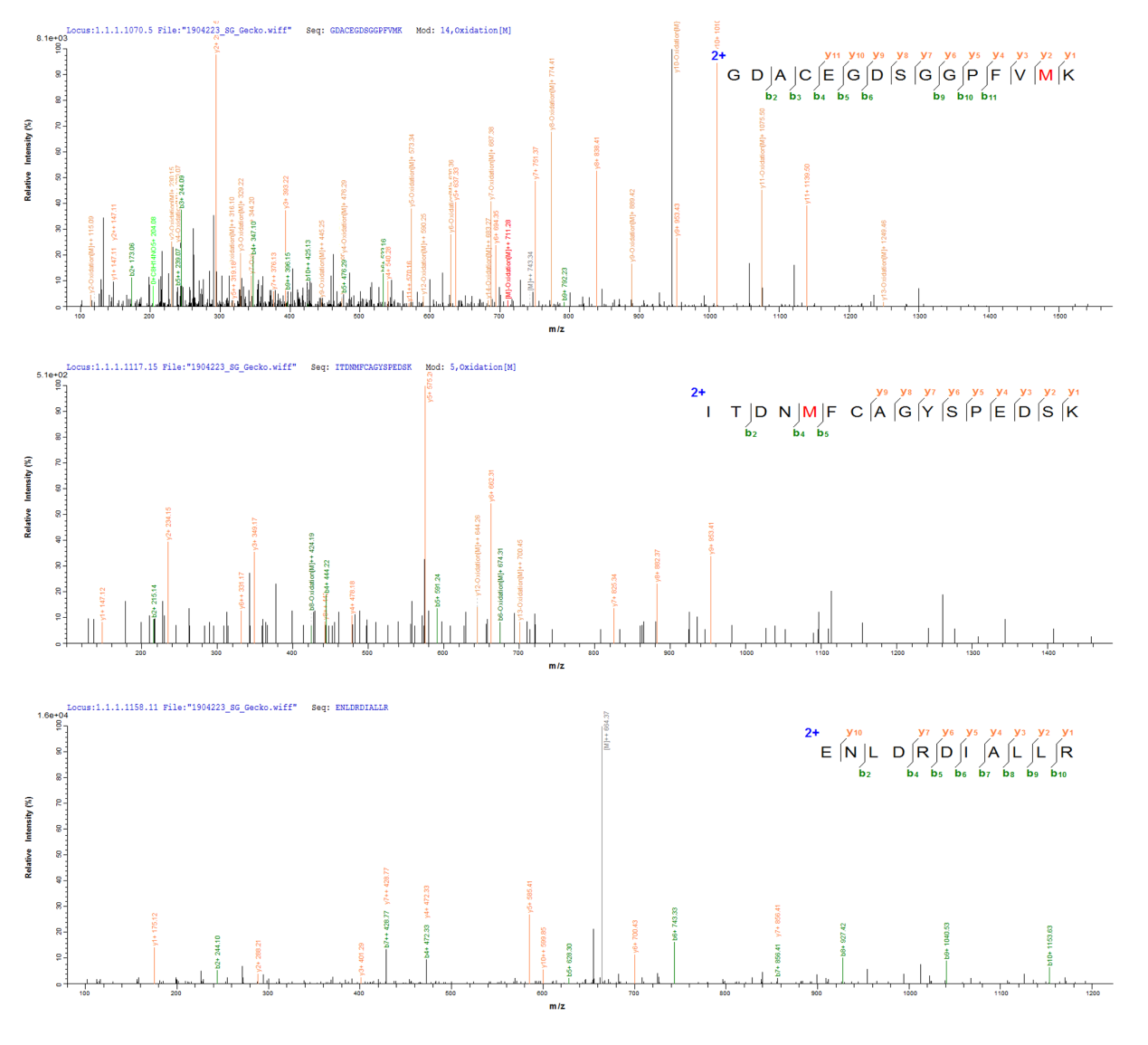


**Fig. S2** Analysis of mass spectrometry for the unidentified protein in the purified gecko thrombin.


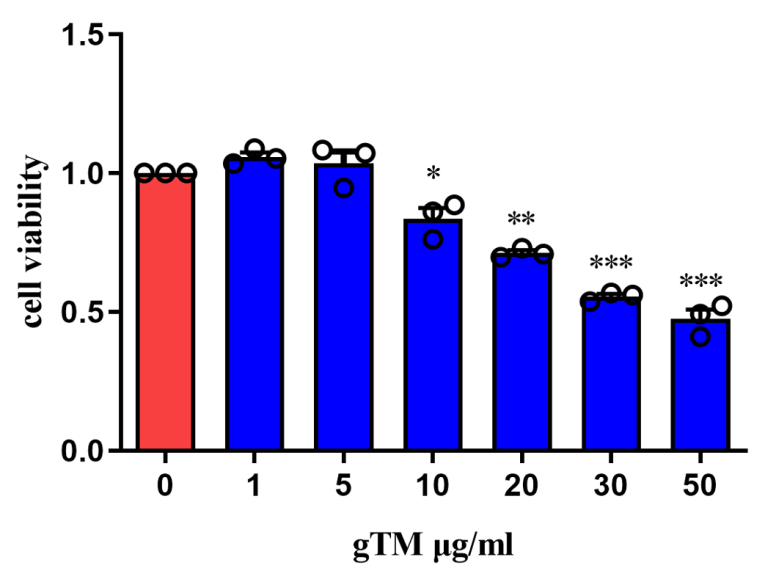


**Fig. S3** CCK-8 assay of cell viability of the gecko oligodendrocytes Gsn3 following treatment of 0-50 μg/ml gTM for 24 h.


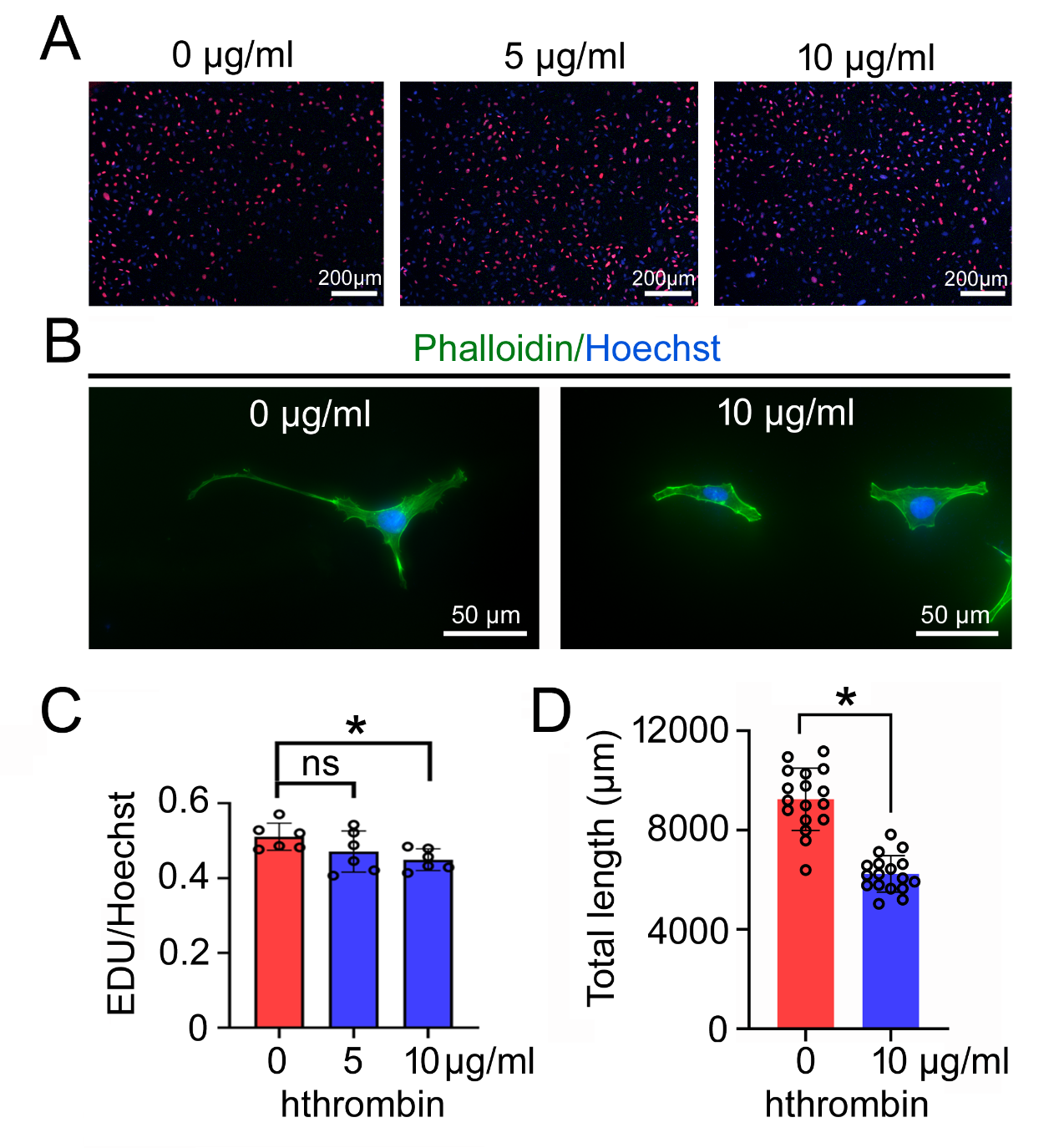


**Fig. S4** Effects of hthrombin on the gecko oligodendrocytes. **A** EdU assay of gecko oligodendrocyte cell line Gsn3 following treatment with 0-10 μg/ml hthrombin for 24 h. **B** Effects of 0-10 μg/ml hthrombin on the process elongation of Gsn3. C Statistical analysis of (A) from six experiments. D Statistical analysis of (B) from seventeen fields each 20 cells. Data are represented as mean ± SEM (P < 0.05). Scale bars, 200 μm in (A); 50 μm in (B).
