# Supplementary material for: Super‐high procoagulant activity of gecko thrombin: A gift from sky dragon
Source: CNS Neurosci Ther. 2023 May 5;29(10):3081–93. doi: 10.1111/cns.14250 (PMC10493662; doi:10.1111/cns.14250)

Full unedited gel/blot for Figure 7

GFAP

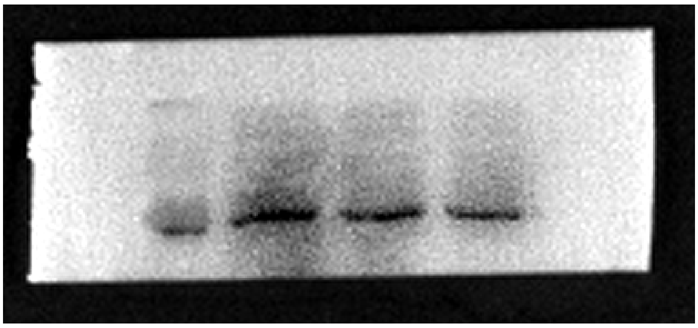

GAPDH

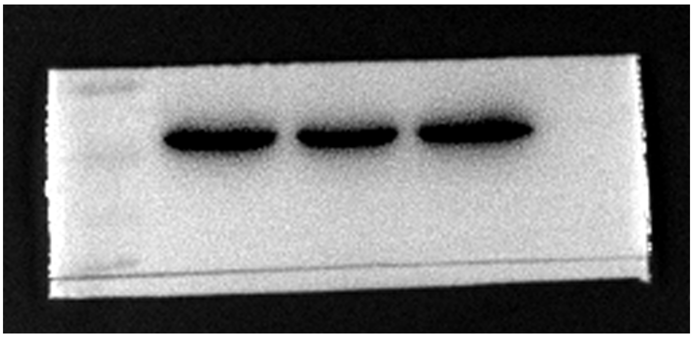

Full unedited gel/blot for Figure 9

p-ERK

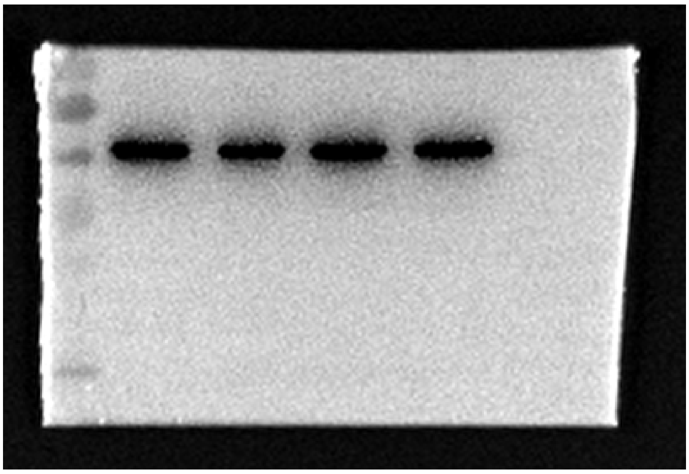

ERK

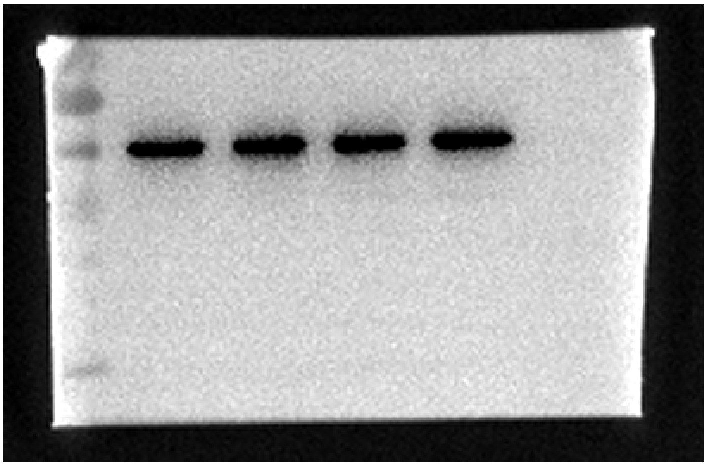

p-P38

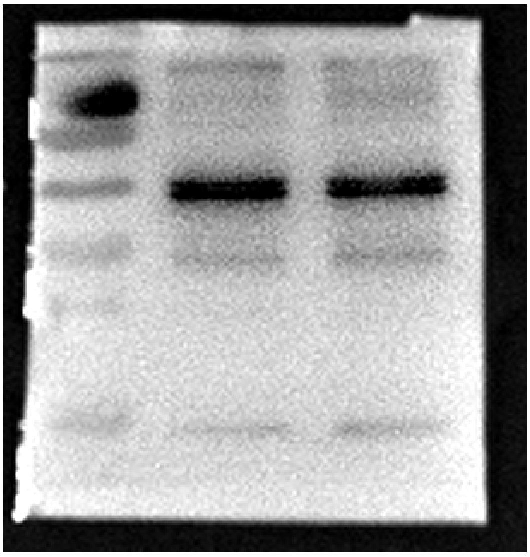

P38

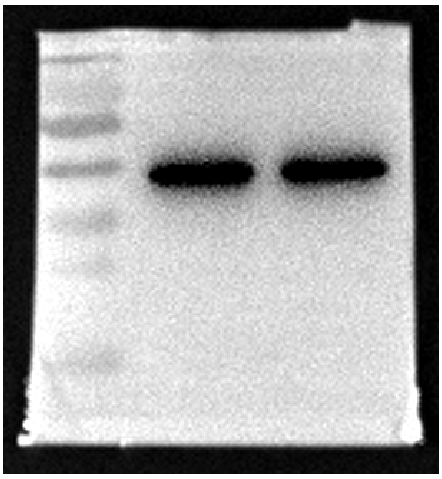

p-JNK

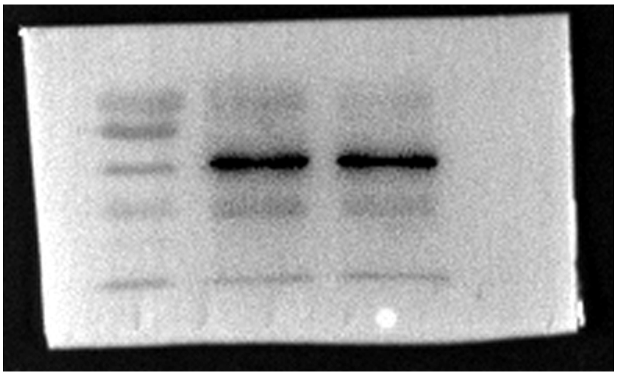

JNK

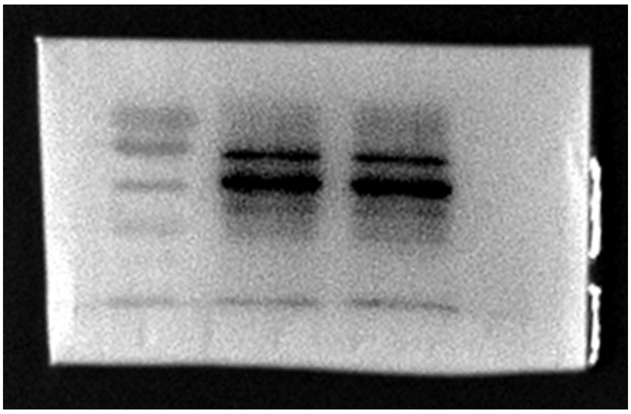

Supplement: Supplementary file 2 — Data S1. [file CNS-29-3081-s002.pdf]
